# Optical imaging of bacterial infections

**DOI:** 10.1007/s40336-016-0180-0

**Published:** 2016-05-04

**Authors:** Bethany Mills, Mark Bradley, Kevin Dhaliwal

**Affiliations:** EPSRC IRC Hub, MRC Centre for Inflammation Research, Queens Medical Research Institute (QMRI), University of Edinburgh, Edinburgh, UK; School of Chemistry, Kings Buildings, University of Edinburgh, Edinburgh, UK

**Keywords:** Optical, Fluorescence, Infection, Bacteria, Imaging, Pre-clinical

## Abstract

The rise in multidrug resistant (MDR) bacteria has become a global crisis. Rapid and accurate diagnosis of infection will facilitate antibiotic stewardship and preserve our ability to treat and cure patients from bacterial infection. Direct in situ imaging of bacteria offers the prospect of accurately diagnosing disease and monitoring patient outcomes and response to treatment in real-time. There have been many recent advances in the field of optical imaging of infection; namely in specific probe and fluorophore design. This combined with the advances in imaging device technology render direct optical imaging of infection a feasible approach for accurate diagnosis in the clinic. Despite this, there are currently no licensed molecular probes for clinical optical imaging of infection. Here we report some of the most promising and interesting probes and approaches under development for this purpose, which have been evaluated in in vivo models within the laboratory setting.

## Introduction

Multidrug resistant (MDR) bacteria are now responsible for upwards of 23,000 deaths per year in the USA and 25,000 deaths per year within the EU; leading to total economic burdens of $35 bn and €1.5 bn, respectively [1, 2]. The inexorable rise of MDR bacteria has in large part been exacerbated by misdiagnosis and inappropriate use of antibiotics [3–6]. Accelerated development of diagnostic approaches is imperative in order to gain control and optimise infection treatment [7]. Improved antimicrobial stewardship will be essential to safeguard not only treatment of bacterial infections, but also to preserve many procedures common to modern medicine where an inability to prevent infection would deem them too risky to perform [8]. These include cancer chemotherapy, dialysis for end stage renal failure, organ and bone marrow transplantation and complex surgery, such as cardiac bypass [1]. On this basis, there is increasing pressure to find methods for the accurate and rapid diagnosis of infection; and in situ molecular imaging presents an ever-attractive tool to achieve this.

### Molecular imaging

Molecular imaging is an approach which, broadly speaking, utilises a functional group to target disease biomarkers or pathways conjugated to an imaging contrast agent, such as a radionuclide or fluorophore. Due to the specificity of these tracers, this approach has the potential to provide a dynamic assessment of a disease site, enabling host–pathogen interactions to be elucidated and the microenvironment to be monitored (e.g. the presence of specific enzymes or pH of the surrounding tissue) rapidly and in real-time. An approach which is much more difficult to achieve using conventional molecular laboratory tests performed ex vivo.

Imaging and sensing infection within the context of the host has the potential to provide answers to fundamental questions, such as:

1. Is the clinical presentation caused by bacteria or sterile inflammation?
2. Are the bacteria Gram positive or Gram negative?
3. Are the bacteria pathogenic; are they MDR?
4. Is the innate host response sufficient to kill and remove the pathogens or should we treat with antimicrobials or other immunomodulatory approaches?
5. Has treatment been effective; should treatment be continued or de-escalated?

To date, direct in situ real-time detection of infection has largely relied upon nuclear imaging of suspected infection with [^18^F]FDG (2-fluoro-2-deoxy-d-glucose) or radiolabelled leukocytes. Despite the high sensitivity of [^18^F]FDG, it has low specificity for bacterial infection and may result in misdiagnosis [9–15]. Conversely, leukocytes radiolabelled with Indium-111, Technicium-99m or [^18^F]FDG should provide enhanced specificity for bacterial infection compared to [^18^F]FDG [16–19], however, in practice specificity has shown to be varied [20, 21]. Other limitations of radiolabelled leukocyte imaging include the lengthy procedure required for retrieving and labelling leukocytes. Additionally, recovering enough leukocytes from immunocompromised patients for the procedure is not always possible, and not all bacterial infections elicit a robust neutrophil response, such as *Mycobacterium tuberculosis*.

Each of these existing ‘gold standard’ imaging techniques rely on imaging surrogates of the response to infection rather than targeting bacteria directly. Hence these methodologies are inherently lacking in ability to determine bacterial load or Gram status. Therefore, there is a concerted effort to develop bacteria specific radionuclide probes [22–33]. These probes are ultimately designed with a target product profile to have high sensitivity and specificity; meaning infection loci can be accurately identified by whole body imaging. These probes also strive to quantify bacterial load, elucidate the Gram status of the bacterial infection and be used to monitor treatment progression. However, there are several fundamental limitations associated with nuclear medicine, which are unlikely to be overcome with new generation bacterial tracers. Namely this is the cost associated with the labelling and synthesis of these probes; their short shelf-life due to decay of the radionuclide; the radiation dose to the patient and medical staff; and that the whole body scanning procedure is not suitable for some patients, e.g. for those in intensive care or in theatres; where near-patient imaging is optimal (some hand held devices are now available, however, the use of radioactivity in complex clinical environments is challenging and not always possible). Therefore, the development of optical molecular imaging tools based on fluorescence reporters is gaining in popularity, especially for superficial surfaces of the human body; such as skin or those accessible by endoscopy. Whilst these probes are designed to stand alone, it is highly likely that these probes would be used in combination with current whole body imaging; for example [^18^F]FDG could be used to determine potential infectious loci, which could then be further investigated and monitored with optical probes. This approach may be clinically important for patients with fever of unknown origin (FUO).

### Optical molecular imaging

Optical molecular imaging relies on the detection of fluorescence at a target site. Despite its widespread use within the laboratory for generating insight into biological processes in small animal models, optical imaging for human diagnostics has not yet widely gained adoption within the clinical setting. This has been for a number of reasons; including poor signal to noise of fluorophores within the body, the lack of specificity and sensitivity of available probes, toxicity, and the inherent issue of light scattering and absorbance within tissues. The current challenges are discussed in further detail by Keereweer [34], thus to date, no optical probes have been licensed for use or extensively evaluated within the clinic for bacterial detection. Despite this, optical imaging has shown its feasibility in the field of oncology where its worth has been demonstrated using intraoperative imaging devices for image guided surgery [35].

Moreover, due to recent advances in both fluorophore design and synthesis, and in imaging technology [36], many of the limiting factors associated with optical imaging can now be addressed and minimised, and on-going efforts towards infection detection in vivo are producing a variety of interesting probe candidates.

There is an ever-increasing number of available fluorophores with desirable characteristics for in vivo and clinical imaging. These fluorophores are shifted towards the near infrared (NIR, 700–900 nm) region of the electromagnetic spectrum [37–39] and therefore have reduced tissue absorbance and scattering, so that they can be detected from greater depths and allow for more sensitive imaging [40–42]. Furthermore, indocyanine green (ICG) which fluoresces in the NIR region has US Food and Drug Administration (FDA) clinical approval and has been evaluated extensively in humans [43].

Whilst shifting excitation and emission spectra towards the NIR region is imperative for enabling sensitive imaging of below the skin surface [42], other considerations for fluorophore development must be considered in order to make them useful. For example: the aqueous solubility, photostability, lack of auto quenching, ease of conjugation to targeting moieties, effect on the targeting moiety, function, biodistribution, and clearance route to name but a few. As shown by examples later within this review, there are several NIR fluorophores which make interesting candidates for optical imaging of bacterial infections in vivo.

The emergence of environmentally sensitive fluorophores and exploitation of FRET (fluorescence resonance energy transfer) systems has also been conducive to the progression of optical imaging probe design. NBD (7-nitrobenz-2-oxa-1,3-diazol-4-y) is one such environmentally sensitive fluorophore [44]; within an aqueous environment, NBD fluoresces weakly, whereas strong fluorescent signal can be detected from hydrophobic regions, such as the cell membrane. It must be noted that due to the short emission wavelength of NBD (*λ*_em max_ = 535 nm), NBD is not a fluorophore suited at all applications due to high tissue autofluorescence at this wavelength, however as discussed later, NBD has been exploited for some applications and can be detected above tissue background using fibred confocal fluorescence microscopy (FCFM) [45]. FRET systems exploit the phenomenon of fluorescence quenching. If a fluorophore is placed in close proximity to a quencher, the quencher is able to accept the energy emitted from the excited fluorophore, preventing fluorescence signal emission from the probe. In the context of optical imaging probes, the fluorophore and quencher are often located either side of a specific peptide sequence. Cleavage of the peptide sequence by a target enzyme releases the quencher from the molecule, thus enabling fluorescent signal to be detected. This approach has been exploited to target several bacteria specific enzymes, such as β-lactamase [46] and micrococcal nuclease [47], as discussed later.

Additionally, the advancement in hand held-devices and optical endomicroscopy (OEM) platforms such as fibred confocal fluorescent microscopes (FCFM) mean that imaging of shallow infections and those in internally accessible areas such as lungs, stomach, gut etc. can be performed at the bedside without the need to move patients to cumbersome equipment, as is required for nuclear imaging and magnetic resonance imaging (MRI).

Due to the lack of reported clinical data for the evaluation of optical bacteria-specific molecular imaging probes, this review will report and comment on some of the most promising optical imaging agents under development, which have thus far been evaluated in vivo in small animal models.

## Current probes undergoing in vivo assessment in small animal models

Probes for optical imaging are dependent on two key factors; the targeting region and the fluorophore (and quencher if applicable). Both of which are crucial for specific, sensitive imaging tools. Whilst this review is primarily focused on optical imaging of bacterial infections in vivo, probe development for the field of molecular imaging has largely been geared towards nuclear imaging. For this reason, where appropriate, examples from nuclear medicine are reported in the context of optical probe design. Table 1 lists optical probes currently under development as discussed within this review, including their key features and models of pre-clinical evaluation.

**Table 1:** Bacteria-specific optical imaging probes evaluated pre-clinically

Probe	Target	Fluorophore/quencher	Model	Bacteria	Limit of detection (CFU)	Max target: non-target	Notes	References
ICG02-UBI_29–41_	Bacterial cell membrane	ICG02	Mouse Axillary fossa	*E. coli, S. aureus, P aeruginosa*	Not assessed	*S. aureus* ~6, *E. coli* ~3.5	Study was done prior to targeted drug delivery	[53]
UBI-10	Bacterial cell membrane	NBD	Ex vivo human lung tissue	*S. aureus*	Not assessed	Not assessed	Bacteria were pre-labelled prior to addition to lung tissue	[45]
ZnO@BSA-PEP-MPA	Bacterial cell membrane	MPA	Mouse Axillary fossa	*S. aureus, B. subtilis*	Not assessed	~7	Study was done prior to targeted drug delivery	[54]
[^111^ln]-DTPA-Cy5-UBI_29–41_	Bacterial cell membrane	Cy5	Mouse Front paw Thigh muscle	*S. aureus*, *K. pneumoniae*	*S. aureus*: 8.0 ± 4.2 × 10^8^ *K. pneumoniae*: 2.5 ± 1.0 × 10^8^	*S. aureus* ~2.4 *K. pneumoniae* ~3.6	Dual optical-SPECT tracer	[55]
Cy5-Zn-DPA	Bacterial cell membrane	Cy5	Mouse Rear thigh muscle	*S. aureus, S. typhimurium*	Not assessed	4.2	Reported 4-fold less probe required for same T:NT ratio compared to PSVue 794	[60]
NIR-Zn-DPA	Bacterial cell membrane	NIR Cyanine dye	Mouse Rear thigh muscle	*S. aureus*	Not assessed	4–8 dependant on experimental set up	Commercially known as PSVue 794	[61, 62]
Squarine-rotaxane- ZnDPA	Bacterial cell membrane	Squarine	Mouse Muscles over tibia bone in rear leg	*S. aureus*	Not assessed	~6		[64]
MDP-2	Maltodextrin transporter	IR786	Rat Rear thigh muscle	*E. coli*	10^5^	~26	Was shown not to accumulate at sites of metabolically inactive bacteria	[71]
Con A-IR750	Bacterial cell wall	IR750	Mice Skin wound Transcutaneously implanted catheter	*S. aureus*	Skin wound: >1 × 10^7^	Catheter: 5×	Probe was added topically and required a wash step.	[81]
Cy5.5-TT	Micrococcal Nuclease (MN)	Cy5.5/ZEN and Iowa Black RQ	Mice Rear thigh muscle	*S. aureus*	Not assessed	4	Probe unlikely to have access to infection site. Cleaved by MN at infection periphery	[47]
*β*-lactamase FRET	*M. tb* β-lactamase	Cy5.5/QSY21 and QSY22	Mice Subcutaneous Pulmonary	*M. tuberculosis*	10^4^	Not calculated		[46]
AF680-Pro-T	Staphylocoagulase	AF680	Mice Endocarditis	*S. aureus*	Not assessed	20–28		[92]
CytoCy5S	Nitroreductase	CytoCy5S	Mouse Rear thigh muscle Liver and spleen Intratumorally	*E. coli* *B. breve* *S. typhimurium*	<5 × 10^6^	Not calculated	Probe was administered IP	[93]
Vancomycin-IRDye800CW	Gram positive cell wall	IRDye800CW	Mice Hind limb intramuscular injection Human cadaver	*S. aureus*	Mice: 6.4 × 10^7^ Cadaver: 1.25 × 10^8^ CFU/mm^2^	4.2 in mice	Biofilms were pre-labelled prior to implanting into cadaver	[103]
M13-SWNT	*E. coli* F’Pili	SWNT	Mice Right rear flank intramuscular	*E. coli*	Not assessed	~2.4		[107]
Anti- *S. aureus*- M13-SWNT	*S. aureus*	SWNT	Mice Right rear flank intramuscular Endocarditis	*S. aureus*	Not assessed.	Intramuscular ~3.4 Endocarditis ~5.7	The target of the anti-*S. aureus* antibody is not disclosed	[107]

Maximum target-to-non-target ratios are reported in original studies as either target-to-normal, target-to-background or fold changes

*CFU* colony forming units. Bacterial abbreviations are as follows: *B. breve* *Bifidobacterium breve; B. subtilis* *Bacillus subtilis*; *E. coli* *Escherichia coli*; *K. pneumoniae* *Klebsiella pneumoniae*; *M. tuberculosis* *Mycobacterium tuberculosis*; *P. aeruginosa* *Pseudomonas aeruginosa*; *S. aureus* *Staphylococcus aureus*; *S. typhimurium* *Salmonella typhimurium*

As is evidenced from Table 1 and the forthcoming discussion of reported molecular probe data, it is challenging to directly compare probe performance. This is due to the variation in study design; including the pathogen chosen, inoculation dose and infection site; the host immune status; the choice of controls; the injected probe route, concentration and determination of when peak signal to noise is likely to be achieved. It must be noted that the variance in study design is not limited to pre-clinical optical imaging, but is also common place in the field of pre-clinical nuclear imaging. Whilst extrapolating information from individual studies in order to compare probes across the field is difficult, the optical probes discussed in the remainder of this review each demonstrate potential for translation to the clinical setting.

### Antimicrobial peptides

Antimicrobial peptides (AMPs) form part of the innate immune response. They are small amphipathic molecules and are able to readily insert into negatively charged bacterial membranes, thus offering a potential means for bacteria specific detection.

Ubiquicidin (UBI) is an AMP which shows much promise for diagnosing infection within the field of nuclear medicine. A number of studies pre-clinically [23–25, 28] and clinically [27] have demonstrated that a Technetium-99m radiolabelled UBI fragment, [^99m^Tc]-UBI_29–41_ was able to specifically target a number of infection types, proving accurate diagnosis for at least 95 % of human patients for diseases including diabetic foot [48], fever of unknown origin [49], osteomyelitis [50] and hip prosthesis [51]. [^99m^Tc]-UBI_29–41_ imaging has also shown promise in monitoring responses to treatment in vivo [23, 26] and clinically [52].

The same UBI_29–41_ fragment was subsequently labelled with NIR dye ICG02 and evaluated in vivo against Gram positive and Gram negative infections. This ligand was shown in vivo to have deep tissue penetration, and fast clearance. Moreover, the probe was able to accumulate at sites of bacterial infection and not at sites of sterile inflammation. Free dye did not accumulate at the infection site, and the probe could be blocked by pre-injections of unlabelled UBI_29–41_ [53].

An alternative approach for utilising the UBI_29–41_ fragment for bacterial imaging saw modification of the peptide structure in a number of ways, resulting in a cyclic form [45]. This led to improved peptide stability and resistance to proteolytic degradation, which had been observed for linear derivations of fluorescently labelled UBI_29–41_. In addition, the modified UBI scaffold was conjugated to the environmental fluorophore NBD (7-nitrobenz-2-oxa-1,3-diazol-4-y), enabling the probe to switch from ‘off’ to ‘on’ when inserted within a bacterial membrane. Despite NBD emitting in the ‘green’ region of the electromagnetic spectrum, using fibred confocal fluorescence microscopy (FCFM) the researchers were able to demonstrate that bacteria were labelled and visible above the level of tissue autofluorescence within ex vivo human lung tissue.

Further to labelling the linear UBI_29–41_ fragment with a radiolabel or a fluorophore, other approaches have been taken in order to multiplex fluorescently labelled UBI_29–41_ with either an antimicrobial for treatment of infection [54], or with a radiolabel for dual nuclear–optical imaging of infection [55].

To demonstrate the theranostic potential of fluorescently labelled UBI_29–41_ in vivo, UBI_29–41_ was conjugated to ZnO-quantum dots and NIR fluorophore MPA (a hydrophilic derivative of ICG) to yield ZnO@BSA-PEP-MPA for diagnosis of both Gram positive and Gram negative infections [54]. ZnO@BSA-PEP-MPA was further modified to incorporate the Gram positive antimicrobial vancomycin (Van). Whilst in vivo imaging of Van@ZnO@BSA-PEP-MPA does not appear to have been performed, the group showed in their murine model that the Van@ZnO@BSA-PEP-MPA probe was more effective at killing *Staphylococcus aureus* compared to free vancomycin alone; demonstrating that their approach was successful for combining non-invasive bacterial infection diagnosis and targeted treatment.

Development of a dual optical–nuclear probe for imaging infection circumvents the potential limitation of relying on optical imaging alone for identifying infectious foci by enabling non-invasive whole body imaging by PET or SPECT. This should identify infection sites independently of tissue depth and autofluorescence, which may be prohibitive for whole body optical imaging. After identification of suspected infection sites, the optical element of the probe can be imaged and monitored during intervention, such as surgical resection or treatment. The effectiveness of this approach has previously been demonstrated clinically for sentinel node biopsy [56, 57].

The potential of dual modality imaging of infection with UBI_29–41_ has been demonstrated in murine models of *S. aureus* (Gram positive) and *Klebsiella pneumoniae* (Gram negative) intramuscular infections, where UBI_29–41_ was dual-labelled with Cy5 and Indium-111 yielding [^111^ln]-DTPA-Cy5-UBI_29–41_ [55]. This probe accumulated at the sites of bacterial infection after intravenous (i.v) injection and could be detected by sequential SPECT and optical imaging.

### Zn-DPA

Zn-DPA (zinc-(II)-dipicolyamine) is positively charged metal complex, and as such, it readily interacts with negatively charged membranes of both Gram positive and Gram negative bacterial cells, but not with the membranes of healthy mammalian cells [58, 59].

A number of differently labelled versions of Zn-DPA have been developed and evaluated in vivo with *S. aureus* infection models; including a Cy5-like-labelled ZnDPA probe [60] and a NIR-cyanine-labelled Zn-DPA [61, 62], the latter of which is now commercially available (Li-Cor Biosciences). Furthermore, Zn-DPA conjugated to squaraine-rotaxane moieties have also been developed in order to increase brightness and circumvent some of the known limitations of carbocyanine (Cy) dyes such as poor photostability [59, 63, 64].

Despite the successful in vivo labelling of bacterial infections in several studies [59–64], it must be noted that a fundamental flaw of Zn-DPA imaging is that Zn-DPA also labels negatively charged membranes of apoptotic and necrotic mammalian cells [65, 66]. This leads to false identification of bacterial infection by labelling sites of sterile inflammation, and thus renders it a non-specific imaging agent [60–62, 64].

### Sugars

Bacteria and mammalian cells utilise different sugars, hence targeting bacteria specific sugar transport systems is a promising approach for probe design. To date, probes targeting maltohexaose, glucose-6-phosphate and sorbitol transport systems have been reported.

Maltohexaose is a sugar utilised by many bacteria [67], it is transported into bacteria via the maltodextrin transporter which is absent from mammalian cells [68, 69]. When labelled with the NIR dye IR786, the maltodextrin-based imaging probe (MDP) was shown to accumulate at sites of *Escherichia coli* infections in vivo and within *S. aureus* biofilms in vitro specifically due to uptake by the maltodextrin transport pathway. Due to the high activity of the bacterial maltodextrin transporter and the hydrophilic nature of MDP (meaning it cannot diffuse through mammalian cell membranes [70]), the probe demonstrated high signal to noise and showed high sensitivity compared to other bacterial targeting agents, including ZnDPA [71]. However, as with all metabolite-based probes, accumulation of the probe is likely to be dependent on the metabolic state of the bacteria.

Glucose-6-phosphate (G6P) is a sugar utilised only by certain bacteria; including *E. coli* and *S. aureus* [72]. It is transported into bacteria via a Universal Hexose Phosphate Transporter (UHPT) which is not expressed at the cell surface of mammalian cells. Therefore, UHPT is an ideal candidate for delivery of targeted bacteria specific probes. The feasibility of targeting UHPT for infection detection has been demonstrated with the G6P analogue [^18^F]FDG-6-P by nuclear imaging techniques [73]. Converting glucose to glucose-6-phosphate is achieved through a very simple hexokinase reaction; therefore, it could be possible to convert fluorescently labelled glucose to fluorescently labelled glucose-6-phosphate. Both environmentally sensitive NBD [74, 75] and NIR [76] glucose derivatives have been reported. Whilst NBD-glucose has been shown to be transported by specifically glucose transporters, the NIR labelled equivalent was not, speculatively due to the larger size of the NIR fluorophore [77]. However, it is not known whether the phosphorylated versions of these probes would be recognised by the UHPT.

Sorbitol is a metabolite utilised by the Gram negative Enterobacteriaceae family of bacteria [78]. These bacteria include *E. coli*, *K. pneumoniae* and *Pseudomonas aeruginosa* and are the main cause of Gram negative bacterial infections in humans. In order to target these bacteria in vivo, [^18^F]FDG was modified to produce 2-[^18^F]-fluorodeoxysorbitol ([^18^F]FDS). The probe was able to specifically differentiate sites of Enterobacteriaceae infection from Gram positive infection and sites of sterile inflammation in a range of infection sites; including brain and lung. Additionally, this probe was able to monitor infection levels during treatment of antibiotic sensitive and resistant *E. coli* infections [79]. The biodistribution and stability of [^18^F]FDS has since been evaluated clinically in a healthy volunteer, and was shown to be consistent with that of murine models [80]. Despite this promising route for targeting Enterobacteriaceae, fluorescently labelled sorbitol derivatives for optical imaging are yet to be reported.

### Concanavalin A nanoprobe

Concanavalin A (Con A) is a lectin-binding protein with a high affinity for cell-surface mannose residues and other polysaccharides within the cell wall of Gram positive and Gram negative bacteria. To exploit Con A as a bacterial imaging tool, Con A was conjugated to a nanoparticle carrier and near NIR dye IR750 [81]. The Con A-IR750 nanoprobe was demonstrated to adhere to sites of superficial *S. aureus* wound site infections, and at sites of infected transcutaneously implanted catheters in mice. For each of these infection models the nanoprobe was added topically followed by a wash step and imaging. Fluorescence signal from the wound site was detected and increased proportionally with bacterial load.

### Nuclease-activated FRET probe

Nucleases are a diverse group of enzymes responsible for the degradation of nucleic acids (through specific cleavage of the phosphodiester bonds between nucleotide subunits). Several modified oligonucleotides have been identified which show resistance to degradation by mammalian nucleases, but are susceptible to cleavage by certain bacterial nucleases [82]. One of the identified oligonucleotides was sensitive to digestion specifically by *S. aureus* Micrococcal Nuclease (MN), but resistant to mammalian serum nucleases [47].

This identified oligonucleotide was further modified to incorporate a FRET (fluorescence resonance energy transfer) system, where a Cy5.5 fluorescence group was incorporated 5′ of the enzyme cleavage site, and quenching groups (ZEN and Iowa Black RQ) 3′ of the cleavage site. The probe was silent until contact and cleavage by MN, enabling identification of *S. aureus* infections in vivo within 15 min of i.v administration.

### β-Lactamase-activated FRET probe

β-Lactamase is an enzyme produced by many bacteria and confers resistance to β-lactam antibiotics, such as penicillin [83–86]. The structure of β-lactamase encoded by *Mycobacterium tuberculosis* (*M. tb*) differs to that produced by other bacterial species and is known to be highly active [87, 88]. It has therefore has been exploited for detection of *M. tb* using a FRET-based NIR (Cy5.5 and QSY21/QSY22) substrate for *M. tb* β-lactamase. A number of probes were evaluated in subcutaneous in vivo murine models of *M. tb* infections [46], with each candidate accumulating and being activated at the site of infection. The probe with the highest sensitivity was selected for further investigation of pulmonary *M. tb* infection. Following i.v administration the probe was able to reach the site of infection, was activated and was detectible in situ with a high signal to noise. Signal was not detected in the lungs of non-infected mice both in situ and ex vivo.

### Prothrombin

A mechanism of *S. aureus* immune evasion is to clot human blood through the binding of staphylocoagulase to prothrombin [89–91]. This phenomenon has been exploited to enable *S. aureus* detection in vivo in mouse models of endocarditis by optical imaging of an Alexa Fluor 680 labelled prothrombin analogue, AF680-Pro-T [92]. Accumulation of the probe at the infection site was shown to be specific to coagulase positive *S. aureus*; with no probe detected at sites of coagulase negative *Staphylococcus epidermidis* infection or non-infected controls.

### Nitroreductase-activated probe

Nitroreductases are widespread throughout both Gram positive and Gram negative bacterial species, and therefore their exploitation through optical imaging would act as a general bacterial infection identifier [93]. This has been demonstrated by using the self-quenched NIR fluorophore CytoCy5S, which is reduced by nitroreductase to yield fluorescence signal [94]. Through this approach it was possible to detect a number of pathogens in different murine infection models in vivo by whole body imaging, including intramuscular *E. coli* infections, liver and spleen *Salmonella typhimurium* infections, and tumour colonisation by *E. coli* and *Bifidobacterium breve*.

### Antibiotics

Historically, there have been numerous attempts at developing antibiotics as bacterial imaging agents [95]. The most widely explored was the commercially available probe Infecton ([^99m^Tc]-ciprofloxacin) [96, 97]. Radiolabelled ciprofloxacin was anticipated to act as a general identifier of Gram positive and Gram negative bacterial infection; however, in vivo investigations and patient clinical studies demonstrated varying degrees of success; indicating that it may not enable a clear distinction between bacterial infection and sterile inflammation [98–101]. Due to the inconclusive data, development of infection was ceased in 2007.

More recently, a NIR-labelled vancomycin probe has begun in vivo evaluation. Vancomycin binds directly to the cell wall of Gram positive bacteria [102], and therefore accumulates at sites of infection. When Vancomycin-IRDye800CW was injected i.v into mice harbouring bilateral *S. aureus* and *E. coli* infections, the probe could be detected selectively in the *S. aureus* infected flank [103]. Moreover, this group also studied the feasibility of using Vancomycin-IRDye800CW to detect Gram positive biofilms on implanted medical devices within human cadavers. Osteosynthetic devices were coated with *S. epidemidis* biofilms pre-labelled with Vancomycin-IRDye800CW, and implanted into lower leg of cadavers. Fluorescence signal was detected through the skin using an intraoperative clinical multispectral imaging camera, whereas no signal was detected for bacteria free controls. It has not yet been determined whether this approach would be suitable for imaging deeper infections within the body, such as hip prosthesis or endocarditis.

A major drawback of targeting bacteria with radiolabelled antibiotics is that bacteria which are already resistant to the drug are unlikely to accumulate the probe, and as such could result in false negative results. Moreover, there are questions surrounding what effect administering sub-lethal doses of antibiotic probe may have on promoting antimicrobial resistance [104]; however, the dose required for imaging will remain low and this is likely to not be an issue.

### Bacteriophage

The bacteriophage M13 (a filamentous, non-lytic phage with natural affinity for the *E. coli* F’ pili [105, 106]) has been exploited for bacterial targeting in vivo. Bacteriophage M13 was used as a scaffold for attaching single-walled carbon nanotubules (SWNTs). These fluoresce in the NIR-II region of the electromagnetic spectrum (900–1400 nm) [107], resulting in reduced tissue scattering and enhanced depth penetration, providing a potential improved signal to noise by 100-fold compared to other NIR probes (700–900 nm) [108–111]. The M13-SWNT probe was able to discriminate between F′ positive and F′ negative *E. coli* soft tissue infections in a mouse model with a custom made NIR-II imaging device. The M13-SWNT probe was further modified to contain an anti-*S. aureus* antibody (anti-*S. aureus*-M13-SWNT), which enabled specific targeting to soft tissue and endocarditis *S. aureus* infections in mice, and demonstrated the potential of bacteriophage-mediated imaging of infections other than *E. coli.*

## Future perspectives

As the global number of MDR pathogens continues to rise, the urgency to answer the question of whether to treat or not, whether treatment is effective, and when to stop treatment on a case-by-case basis is imperative. To this end, a range of bacterial probe targets have been identified and the approaches under development to image these are varied. Whilst the emphasis in recent years has been to develop tools for specifically imaging infection by nuclear medicine, the cost, short shelf-life and radiation dose exposure are all undesirable characteristics inherent to this approach. The expanding field of optical probe design presents an alternative means to detect infections in situ whilst circumventing many of the drawbacks of nuclear imaging. Whilst optical imaging approaches do have several potential limitations; such as low sensitivity, poor depth penetration and unavoidable tissue autofluorescence; advances in detection technology and the availability of self-quenched, FRET-systems, environmentally activated, and ‘always-on’ fluorophores with distinct spectral properties could minimise these limitations and offer advantages for multiplexing probes and improve signal to noise compared to ‘always-on’ radionuclides.

It is clear that infection specific molecular probes for both nuclear and optical imaging modalities should continue to be developed in order to solve the unmet clinical need for rapid and accurate in situ diagnostics of a range of bacterial species. It is unlikely that whole-body optical imaging will ever be feasible to identify unknown infection loci sites. For this type of investigation, radiolabelled probes offer a clear advantage. Despite this, optical probes my offer advantages during surgical intervention, identifying prosthetic joint infections or in locations accessible by optical endoscopes, such as for pulmonary infection.

There are currently no molecular probes licensed for clinical optical imaging of infection. However, the field of optical imaging of infection is rapidly expanding and is poised for evaluation of lead probes in first-in-man clinical studies within the coming years; where on-target labelling, ability to target MDR bacteria, ability to target bacteria within a biofilm, probe sensitivity limits and probe biodistribution will be evaluated. We predict that the likeliest probes to be accelerated into clinical practice will be fluorescently labelled antibiotic derivatives and modified antimicrobial peptides. The versatility of optical molecular imaging of bacterial infections is immense and the potential to multiplex to image host–pathogen interactions will lead to a new generation of biological insights.
